# Phyllosphere microbiome responses to nano-berberine and chemical fungicides in powdery mildew infected strawberry

**DOI:** 10.3389/fpls.2025.1712123

**Published:** 2025-12-08

**Authors:** Meng Yang, Tao He, Romy Moukarzel, Man Li, Mei Li, Zengxiu Zhang, Yiyi He, Yixiang Liu, Lei Yu, Shusheng Zhu, Fei Du

**Affiliations:** 1 State Key Laboratory for Conservation and Utilization of Bio-Resources in Yunnan, Yunnan Agricultural University, Kunming, China; 2 Key Laboratory for Agro-Biodiversity and Pest Control of Ministry of Education, Yunnan Agricultural University, Kunming, China; 3 Department of Pest-management and Conservation, Lincoln University, Lincoln, New Zealand; 4 College of Agronomy/Yunnan Urban Agricultural Engineering & Technological Research Center, Kunming University, Kunming, China

**Keywords:** strawberry, powdery mildew, nano-berberine, phyllosphere microorganism, *Bacillus siamensis*, plant microb interaction, microbial ecology, foliar pathogens

## Abstract

Strawberry powdery mildew, caused by the obligate biotroph *Podosphaera aphanis*, is a major threat to commercial strawberry production, reducing both yield parameters and fruit quality. While chemical fungicides remain a standard control method, their non-target effects on phyllosphere microbial communities have raised important ecological and environmental concerns. Nano-pesticides are increasingly applied in plant disease management, however, their influence on the composition and functional potential of phyllosphere microbial communities remains poorly understood. The nano-berberine formulation (BBR-M) used in this study was provided by a collaborative group, with synthesis and physicochemical characteristics consistent with those previously reported for this material. In this study, we compared the field-level effects of a nano-berberine formulation (BBR-M) and conventional chemical fungicides (e.g., bupirimate) on the strawberry phyllosphere microbiota using high-throughput sequencing, bioinformatics analysis, and microbial isolation techniques. The results showed that nano-fungicide application significantly reduced the disease index of powdery mildew and markedly decreased its incidence in field-grown strawberries, ultimately lowering leaf disease incidence to 5.06% with a control efficacy of 96.81%. Furthermore, nano-fungicides and conventional chemical fungicides treatments were associated with distinct impacts on the phyllosphere microenvironment of strawberry. Application of BBR-M was associated with a more structured and potentially stable microbial community, characterized by increased fungal diversity and higher modularity in co-occurrence networks. In contrast, bupirimate treatment increased microbial complexity but coincided with reduced network stability. A strain of *Bacillus siamensis*—a genus identified as a core taxon within the BBR-M phyllosphere network—was subsequently isolated from nano-berberine–treated leaves and exhibited strong antagonistic activity against *Colletotrichum nymphaeae*. Field assays showed that this strain effectively suppressed strawberry powdery mildew with 98.18% control efficacy. Collectively, these findings provide important insights into the ecological safety and functional implications of novel pesticide technologies, underscoring the potential of nano-fungicides and native biocontrol agents for sustainable strawberry disease management.

## 1 Introduction

Strawberry (*Fragaria × ananassa* Duch.) is a high-value horticultural crop widely cultivated in protected facilities such as solar greenhouses and plastic tunnels in China (Afrin et al., 2016). These enclosed conditions characterized by high humidity and temperature, coupled with continuous cropping, favor pathogen accumulation and frequent disease outbreaks such as powdery mildew, gray mold, and leaf spot (Claire et al., 2018; Petrakis et al., 2024). Powdery mildew, caused by *Podosphaera aphanis*, remains one of the most prevalent foliar diseases, seriously affecting yield and fruit quality, which can cause yield losses exceeding 50% (Aldrighetti and Pertot, 2023).

For decades, chemical fungicides have remained the primary strategy for managing powdery mildew, despite growing concerns regarding their environmental impact and long-term sustainability (Carisse et al., 2013; Dong et al., 2023; Yan et al., 2021). Excessive and prolonged use of fungicides not only accelerates pathogen resistance but also disturbs non-target microbial communities in the phyllosphere and rhizosphere, leading to reduced soil enzyme activity, altered redox balance, and inhibited microbial diversity (Goyal et al., 2023; Ke et al., 2021; Shahbazi et al., 2014). Among them, bupirimate-a systemic pyrimidine fungicide widely used to control strawberry powdery mildew-acts by inhibiting adenosine dehydrogenase in the pathogen, disrupting metabolic processes and causing spore mortality (Chen et al., 2023a, b). Due to its broad-spectrum activity and low toxicity, bupirimate remains a benchmark in commercial production, yet its effects on phyllosphere microbial composition and function remain poorly understood.

Recent research has shown that nanoparticles (NPs) can interact with plant-associated microbiota, influencing community structure and function through changes in nutrient cycling, redox potential, and enzyme activity (Cao et al., 2024; Li et al., 2023). Nanoparticles have been applied both to roots and leaves, affecting microbial colonization and nutrient mobilization in complex ways. Compared to conventional formulations, nano-enabled agrochemicals offer controlled release, enhanced solubility, and potentially lower non-target toxicity (Jali et al., 2024). For instance, chitosan-based nanoparticles have been reported to enhance disease resistance in rice by modulating the rhizosphere and phyllosphere microbiomes, maintaining beneficial bacterial and fungal taxa while suppressing pathogens (Hafeez et al., 2024).

Berberine (BBR), an alkaloid with a ubiquitous presence in traditional Chinese medicinal plants such as *Coptis chinensis* and Amur cork-tree, has found extensive applications across both pharmaceutical and agricultural domains. Its broad-spectrum antimicrobial efficacy has been empirically substantiated through experimental research (Guo et al., 2020; Habtemariam, 2016; Liang et al., 2022; Liu, 2023). Nano-berberine are produced by integrating the self-assembly of small molecules (SMSA) with berberine (BBR) and curcumin (CM). This nano-fungicide can utilize natural resources like natural fungicides, sunlight, and oxygen. Additionally, its preparation process is straightforward and efficient, making it promising for sustainable development (Tian et al., 2022). However, it is not yet clear whether this novel green nano-fungicide can reduce non-target impacts on strawberry leaf surface microbiota.

Similar to other plant species, strawberry hosts a highly diverse community of prokaryotic and eukaryotic microorganisms on its phyllosphere (Martin et al., 2017). These microbes form complex interaction networks that play essential roles in promoting plant productivity and maintaining overall plant health under natural conditions (Li et al., 2022; Lutz et al., 2023; Vogel et al., 2021; Xu et al., 2022; Yin et al., 2021). Beneficial microorganisms contribute to disease suppression through various mechanisms, including induction of plant resistance, nutrient competition, hyperparasitism, and the production of antimicrobial compounds. These processes are interrelated and collectively shape the structure and function of the phyllosphere microbiome (Köhl et al., 2019). Emerging evidence highlights that the phyllosphere harbors functionally important microbial communities capable of suppressing bacterial pathogens. Reconfiguration of the phyllosphere microbiome may lead to the enrichment of beneficial taxa that enhance plant growth, improve nutrient uptake efficiency, and strengthen resistance to abiotic stress and disease. These microbe-mediated interactions ultimately contribute to improved plant health and productivity (Castrillo et al., 2017; Liu et al., 2023). As such, the targeted manipulation of core phyllosphere microbiota has become a promising strategy for foliar disease control and maintaining microbial homeostasis in sustainable agricultural systems (Fitzpatrick et al., 2018). In the rice phyllosphere, the symbiotic fungus *Aspergillus cvjetkovicii* has been reported to secrete a small-molecule signaling compound, 2,4-di-tert-butylphenol (2,4-DTBP), which suppresses the virulence of pathogenic fungi such as *Rhizoctonia solani* Kuhn (teleomorph: *Thanatephorus cucumeris* (Frank) Donk) and *Fusarium fujikuroi* (Fan et al., 2024). In tomato, infection by *Pseudomonas syringae* pv. *tomato* (Pst DC3000) has been shown to selectively promote the enrichment of the beneficial bacterium *Bacillus subtilis* in the rhizosphere and stimulate biofilm formation, thereby contributing to the restructuring of the phyllosphere microbial network (De Coninck et al., 2015; Rudrappa et al., 2008).

Traditional microbiological techniques such as tissue culture have long been used to explore microbial physiology, metabolism, and plant–microbe interactions. However, these methods offer limited insight into the complexity of microbial networks at the microscale. The advent of high-throughput sequencing and bioinformatic tools has opened new avenues for examining the ecological impact of chemical agents on microbial communities. Changes in microbial diversity and interaction networks can markedly influence plant disease resistance and productivity. However, it remains unclear whether nano-fungicides such as nano-berberine and conventional fungicides like bupirimate differentially reshape the phyllosphere microbiome of strawberry, and how these changes affect microbial community function and ecological balance. Clarifying these distinctions is essential for evaluating the ecological compatibility and sustainable application potential of nano-fungicides.

We hypothesized that nano-fungicides would offer effective disease suppression comparable to chemical fungicides, while causing less disturbance to the structure and function of phyllosphere microbial communities. To test this hypothesis, we aimed to: (1) evaluate the field efficacy of nano-fungicides and conventional chemical fungicides in controlling strawberry powdery mildew; (2) investigate the effects of these treatments on the restructuring of phyllosphere microbial communities using high-throughput sequencing; and (3) isolate beneficial bacterial strains from the phyllosphere and assess their biocontrol activity against powdery mildew.

## 2 Materials and methods

### 2.1 Site description

The field experiment was conducted using the strawberry cultivar ‘Monterey’. Treatments were applied on May 30, 2024, at the Strawberry Science Demonstration Base located in Daibu Town, Huize County, Qujing City, Yunnan Province, China (103°24′10.538″ E, 26°10′9.842″ N; elevation 2,509 m). The site has been under continuous strawberry monoculture for six consecutive years and was previously fumigated with 98% dazomet. Strawberries were grown on raised beds under standard cultivation practices. Prior to treatment, no visible disease symptoms were observed, and no fungicides had been applied. To minimize external interference, the experimental plot was physically isolated from adjacent plots using plastic film barriers, and buffer rows were established to reduce edge effects. The composite soil at the site had a pH of 7.00. Throughout the experimental period, environmental conditions were monitored, with an average temperature of 18.58°C, relative humidity of 80.30%, and precipitation of 2.15 mm.

### 2.2 Experimental design

Nano-berberine (BBR), defined as the B-C SMPs from our collaborative study (Tian et al., 2022), synthesized at 25°C and pH 5.0 with a BBR to CM molar ratio of 2:1, exhibited the following key properties: spherical morphology, 407 nm mean particle size, 0.283 polydispersity index, and +24.4 mV zeta potential. It was provided by China Agricultural University, while the 25% emulsifiable concentrate (EW) of bupirimate was sourced from Jiangxi Heyi Chemicals Co., Ltd. The experimental treatments included: (1) three nano-berberine concentrations 2000 mg/L (BBR-L), 2500 mg/L (BBR-M), and 3000 mg/L (BBR-H); (2) a conventional chemical fungicide treatment of 25% EW bupirimate diluted 216-fold (BPM); and (3) a control group treated with sterile water (CK).

Each treatment was applied to a total of 45 strawberry plants, arranged as three biological replicates, each containing 15 plants. The experiment comprised five treatments, resulting in a total of 15 plots, which were arranged in a randomized complete block design. Fungicide applications were performed at 7-day intervals using a pressurized backpack sprayer, with a total of three applications. Spraying was performed at 4:00 PM, ensuring uniform coverage of both the upper and lower leaf surfaces until slight runoff was observed. Meteorological conditions were recorded at each spraying day. Baseline disease severity was assessed prior to the first application (0 days) by recording disease index and incidence at both the plant and leaf levels. Subsequent assessments were conducted before each application (7 d, 14 d, and 21 d) and 7 d after the final treatment (28 d). For each assessment, five plants per plot (i.e., per replicate) were randomly selected, and all leaves were examined. Disease severity was scored based on a standardized leaf rating scale, and disease index, plant incidence, and leaf incidence were calculated to evaluate the efficacy of each treatment against strawberry powdery mildew.

Disease severity was assessed according to the current national standard for fungicides against powdery mildew of strawberry(https://std.samr.gov.cn/). Leaf-level disease was classified into six grades based on the proportion of the leaf area covered by lesions. 0: no disease; 1: lesions covering less than 5% of the leaf area; 3: lesions covering 6%–15% of the leaf area; 5: lesions covering 16%–25% of the leaf area; 7: lesions covering 26%–50% of the leaf area; 9: lesions covering more than 51% of the leaf area.


$$\begin{array}{l} \text{Disease index}(\%)=100\\ \times \frac{\Sigma (\text{number of diseased leaves at all levels}\times \text{Representative value at all levels})}{\text{total leaves investigated}\times \text{Highest representative value}} \end{array}$$

$$\begin{array}{l} \text{Disease Index Growth Rate}(\%)=100\\ \times \frac{\text{Disease Index after treatment }-\text{ Disease Index before treatment}}{\text{Disease Index after treatment}} \end{array}$$

$$\begin{array}{l} \text{Control Effect }(\%)=100\\ \times \frac{1-(\text{Pretreatment Disease Index of treated plot}\times \text{Post}-\text{treatment Disease Index of treated plot})}{\text{Pre}-\text{treatment Disease Index of control plot}\times Post-\text{treatment Disease Index of control plot}} \end{array}$$

### 2.3 Sample collection

Leaf samples were collected 24 h after the second field application from three treatments: the control (CK), medium-concentration nano-berberine (BBR-M), and conventional chemical fungicide (BPM). This time point was selected to capture the Initial disturbance of the phyllosphere microbiota, following the established methodology from our previous study (Berg and Koskella, 2018; He et al., 2024). The sampling procedure was adapted from the company’s technical specifications and refined according to laboratory conditions. To ensure uniformity, leaves were collected from plants with the same east-facing orientation, at similar heights, and with similar levels of greenness and tenderness.

Each treatment was sampled in six replicates. Leaves were gently collected to avoid damage, labeled, and transferred into sterile 50 mL centrifuge tubes, which were immediately placed on dry ice and transported to the laboratory. Tubes were weighed before further processing.

For microbial elution, potassium phosphate buffer (0.1 mol/L, pH=8.0) was added to each sample at a ratio of 10 mL/g of leaf tissue under sterile conditions in a laminar flow hood. Samples were sonicated for 15 min at 40 kHz, followed by vortexing for 10 s at 20°C and 200 r/min. The washing process was repeated twice. The eluates were combined and centrifuged at 13,000 r/min for 10 min at 4°C. The supernatant was discarded, and the pellet was retained for downstream analysis (Bodenhausen et al., 2013).

### 2.4 Total DNA extraction and PCR amplification of strawberry phyllospheric microorganisms

Microbial DNA was extracted from the collected pellets using the Fast Pure^®^ Soil DNA Isolation Kit (Magnetic Bead) (MJYH, Shanghai, China) according to the manufacturer’s protocol. DNA purity and concentration were assessed using 1% agarose gel electrophoresis and a NanoDrop 2000 spectrophotometer (Thermo Fisher Scientific, USA).

For bacterial community analysis, the V3–V4 hypervariable region of the 16S rRNA gene was amplified using primers 338F (5′-ACTCCTACGGGAGGCAGCAG-3′) and 806R (5′-GGACTACHVGGGTWTCTAAT-3′). For fungal community analysis, the internal transcribed spacer (ITS) region was amplified with primers ITS1F (5′-CTTGGTCATTTAGAGGAAGTAA-3′) and ITS2R (5′-GCTGCGTTCTTCATCGATGC-3′) (He et al., 2024). All primers were synthesized by Shanghai Majorbio Bio-pharm Technology Co., Ltd. (Shanghai, China).

Each 20 μL PCR mixture consisted of 10 μL 2× Pro Taq Master Mix, 0.8 μL of each primer (5 μM), 10 ng of template DNA, and ddH_2_O to a final volume of 20 μL. A no-template control (NTC) with sterile water replacing the DNA template was included in each PCR run to monitor for potential contamination. The PCR conditions were as follows: 95°C for 3 min; 30 cycles of 95°C for 30 s, 55°C for 30 s, and 72°C for 45 s; with a final extension at 72°C for 10 min. The absence of amplification products in the NTCs was verified by 2% agarose gel electrophoresis prior to pooling the individual sample amplicons for sequencing. The PCR products were purified, pooled, and sequenced by Shanghai Majorbio Bio-pharm Technology Co., Ltd.

### 2.5 Isolation of culturable microorganisms and evaluation of pathogen control by *Bacillus siamensis*

Phyllosphere bacteria were isolated using standard pre-treatment methods on nutrient agar solid medium (NA). Antagonistic activity was assessed against *Colletotrichum nymphaeae* strain “4-1,” a primary fungal pathogen of strawberry. A 6 mm mycelial plug of the pathogen was placed at the center of an NA plate, and four bacterial isolates were inoculated approximately 4 cm away in the four cardinal directions. Each plate was incubated at 25°C for 5 d. Antagonistic effects were evaluated by measuring the inhibition zone width and inhibition radius. Inhibition zone width was defined as the distance between the edges of the fungal and bacterial colonies, while inhibition radius was measured from the edge of the fungal colony to the center of the bacterial colony. Inhibition rates were then calculated accordingly.


$$\begin{array}{l} \text{Inhibition rate}(\%)\\ =100\times \frac{\text{colony radius of control}-\text{colony radius in dual culture}}{\text{colony radius of control}} \end{array}$$

The antagonistic strain with the strongest inhibitory effect (such as BS) was selected for molecular identification. The 16S rRNA gene of BS was amplified using the universal bacterial primers 27F (5′-AGAGTTTGATCCTGGCTCAG-3′) and 1495R (5′-GGTTACCTTGTTACGACTT-3′) (Zhang et al., 2022). PCR products were sequenced by Beijing Tsingke Biotech Co., Ltd., and the resulting sequence was compared with known sequences in the GenBank database using BLAST to determine the taxonomic identity of strain BS.

For field validation, BS bacterial suspension was adjusted to an OD_600_ of 0.6 and applied to strawberry plants naturally infected with powdery mildew, with sterile water as the negative control (CK). Each treatment was applied to 15 strawberry plants per plot with three replicates. The suspension was sprayed every 7 days using a pressurized backpack sprayer, for a total of four applications. As previously described, spraying was conducted at 4:00 PM, covering both adaxial and abaxial leaf surfaces evenly until droplets began to form. Meteorological data were recorded on each application day. Prior to each application, five plants per plot were randomly selected, and all leaves were surveyed. Disease index, plant incidence, and leaf incidence were calculated, and pre-treatment disease severity was recorded as the baseline for evaluating control efficacy.

### 2.6 Bioinformatics and data analysis

Raw sequencing reads were processed using a standardized amplicon analysis workflow. Quality filtering was performed with fastp (v0.19.6) (Bastian et al., 2009), and paired-end reads were merged using FLASH (v1.2.7) (Tanja and Steven, 2011). Amplicon sequence variants (ASVs) were inferred via the DADA2 (Edgar, 2013)algorithm implemented in QIIME2 (v2022.2) (Barberán et al., 2012), including chimera removal and denoising. Sequencing was conducted on the Illumina platform with a read length of PE300. The insert size was approximately 468 bp for bacterial 16S rRNA and 350 bp for fungal ITS1 amplicons. Taxonomic classification of bacterial ASVs was performed using the Bayesian classifier against the SILVA 138/16S_Bacteria database with a confidence threshold of 0.7. ASVs assigned to chloroplast and mitochondria were removed prior to downstream analysis. Fungal ASVs were taxonomically annotated using the Bayesian classifier with the UNITE 9.0/ITS_Fungi reference database (confidence threshold = 0.7) in QIIME2. Alpha diversity metrics, including Chao1 richness and Shannon diversity indices, were calculated using Mothur (v1.42.3) (Schloss et al., 2009).

Group-level comparisons of alpha diversity across more than two treatment groups were assessed using the Kruskal-Wallis test; where significant, *post-hoc* pairwise comparisons were performed using the Wilcoxon rank-sum test with Benjamini–Hochberg false discovery rate (FDR) correction. For direct two-group comparisons (e.g., treatment A vs treatment B at a single time point), the Wilcoxon rank-sum test was applied. All tests for alpha diversity were implemented in R (v4.2.2) using the ‘stats’ and ‘rstatix’ packages.

Beta diversity was calculated using Bray-Curtis dissimilarity and visualized by principal coordinates analysis (PCoA). Differences in community composition among multiple treatment groups were tested primarily using PERMANOVA (adonis2 function in the vegan R package) with 999 permutations, and ANOSIM was used as a complementary non-parametric method to corroborate PERMANOVA results (both based on Bray–Curtis distances). Pairwise PERMANOVA comparisons (with p-value adjustment) were performed when required to identify specific group differences.

LEfSe analysis (LDA > 4.0, *p < 0.05*) was employed to identify differentially abundant taxa among treatment (Segata et al., 2011). Venn diagrams were constructed using EVenn (http://www.ehbio.com/test/venn/#/) (Yang et al., 2024).

Microbial functional predictions for bacterial communities were inferred using PICRUSt2 and mapped to KEGG pathways; statistical comparisons of predicted pathway abundances among groups were performed using Kruskal–Wallis tests followed by pairwise Wilcoxon tests with FDR correction. Fungal trophic modes were assigned using FUNGuild, and changes in trophic mode relative abundances among treatments were tested with Kruskal–Wallis and *post-hoc* Wilcoxon tests.

Microbial co-occurrence networks were constructed using the SparCC algorithm (implemented in Python/R) to estimate correlations; edges were retained for |r| > 0.6 and p < 0.05 based on permutation testing (Barberán et al., 2012). Network topology parameters (node number, average degree, modularity, natural connectivity) were computed in Gephi (v0.10.0) and compared among treatments using permutation tests or Kruskal–Wallis where appropriate (Bastian et al., 2009). Where network stability comparisons were performed, we used permutation-based node removal simulations to quantify natural connectivity loss and compared the area under the natural-connectivity decay curves among treatments.

For the disease control efficacy data, ANOVA was conducted using SPSS (v26.0). Significant differences among treatments were determined using the Waller-Duncan multiple range test at the 0.05 probability level.

For the infection data (leaf- and plant-level incidence), a generalized linear model (GLM) with binomial logistic regression was applied in SPSS. The analysis was performed using a Type III model with a 95% confidence level, Wald chi-square statistics, and Wald confidence intervals to estimate the significance of treatment effects. Pairwise comparisons of estimated marginal means were performed using the Least Significant Difference (LSD) method (*p <* 0.05), without Bonferroni adjustment, to determine significant differences among treatments. Data were expressed as the ratio of infected to total leaves (or plants) per replicate.

GraphPad Prism (v8.0.2) was used to generate visualizations of alpha diversity indices, relative abundances of major taxa, and network stability metrics for each treatment group. The raw sequencing reads were deposited into the NCBI Sequence Read Archive (SRA) database (Accession Number: SRP604122, SRP604150).

## 3 Result analysis

### 3.1 Efficacy of different treatments on strawberry powdery mildew

**Figure 1:**
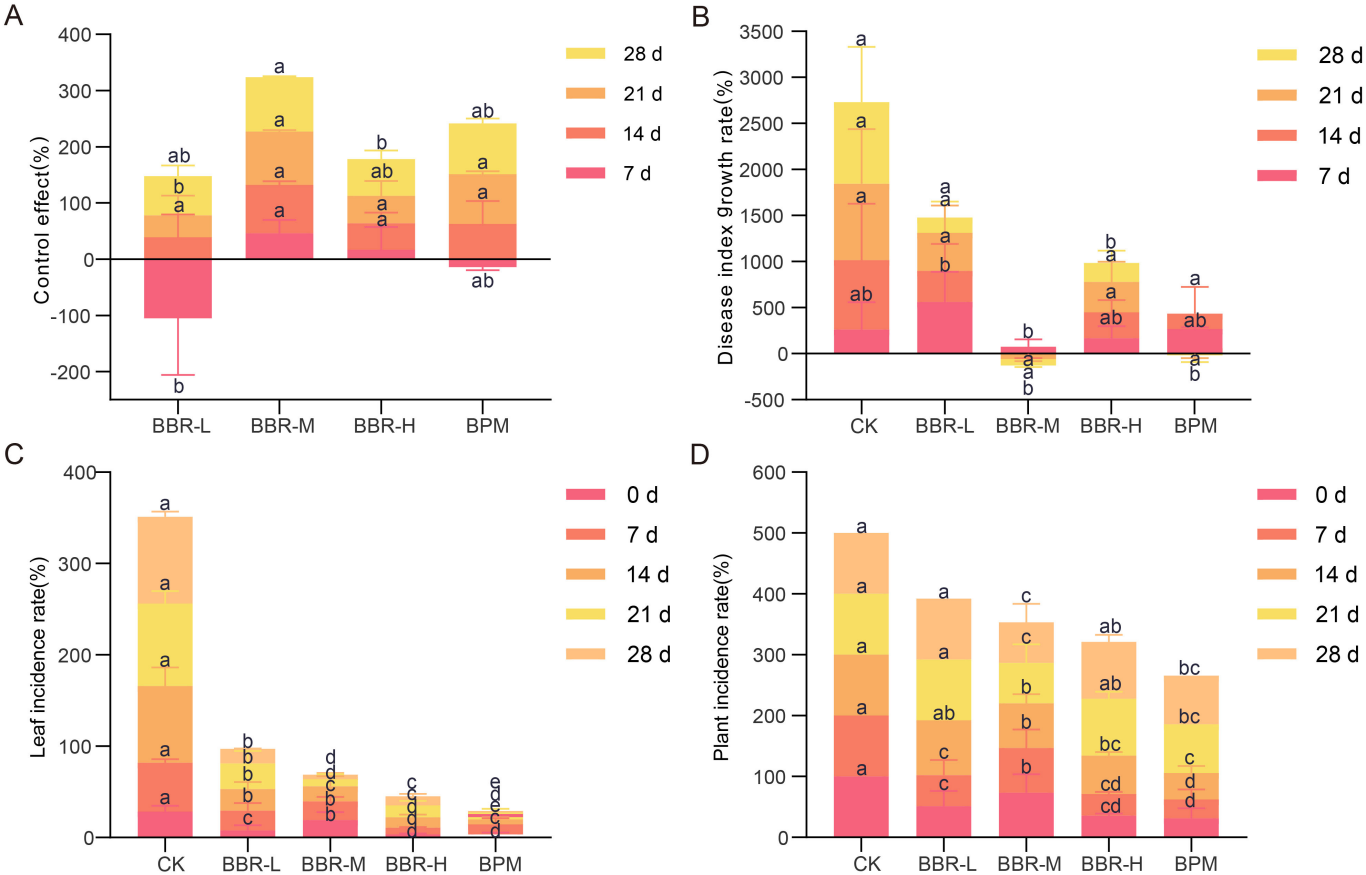
Control effect of strawberry powdery mildew under different treatments. Treatments include control (CK), nano-berberine (BBR-M), and bupirimate (BPM). **(A)** Control efficacy of the first pesticide application measured at 7, 14, 21, and 28 days after spraying; **(B)** Growth rate of disease index on strawberry leaves from 7 to 28 days after the first application; **(C)** Leaf-level incidence of powdery mildew from 0 to 28 days; **(D)** Plant-level incidence of powdery mildew from 0 to 28 days. Bars represent mean ± standard error (n = 3). Control efficacy data were analyzed using one-way ANOVA followed by Waller-Duncan multiple range tests (p < 0.05). Leaf- and plant-level incidence data were analyzed using a binomial generalized linear model (GLM, Type III, Wald test) with pairwise comparisons of estimated marginal means (LSD, p < 0.05). Different lowercase letters indicate significant differences among treatments at the same time point.

The results revealed significant differences in disease control efficacy among the treatments, with BBR-M demonstrating the most effective suppression of strawberry powdery mildew (*p* < 0.05; **Figure 1A**). Analysis of the disease index growth rate across the four spraying intervals highlighted the superior performance of BBR-M, with values declining from 75.12% at 7 days to -71.74% by 28 days post initial application. Although BPM also reduced disease progression, its final growth rate of -16.43%, indicated a weaker and less durable effect compared to BBR-M (**Figure 1B**). Other treatments, including BBR-L and BBR-H, resulted in only marginal reductions or slight increases in disease severity over time. Statistical analysis of disease incidence further confirmed that both BBR-M and BPM significantly reduced infection at both the leaf and plant levels, whereas BBR-L and BBR-H treatments failed to provide consistent suppression (**Figures 1C–D**).

The findings indicate that BBR-M is the optimal concentration of nano-berberine for effective control of strawberry powdery mildew. Therefore, BBR-M was selected for subsequent analysis to compare its effects on the phyllosphere microbial network with those of the conventional fungicide bupirimate. Effects of different treatments on strawberry phyllosphere microbiota.

Alpha diversity analysis of samples collected 24 h after fungicide application showed that the library coverage for all treatments exceeded 99.95%. The rarefaction curves for each sample approached a plateau, indicating that the sequencing depth was sufficient to capture the majority of microbial diversity within each community.

#### 3.1.1 Alpha and beta diversity

Distinct differences in phyllosphere microbial alpha diversity were observed among treatments, all of which showed an increasing trend compared with the control (CK). At the amplicon sequence variants (ASVs) level, within-group comparisons indicated that BBR-M treatment had minimal impact on bacterial diversity but significantly increased fungal diversity (Shannon index: CK/BBR-M = 1.65/2.89). In contrast, BPM treatment significantly enhanced both bacterial and fungal diversity, with Shannon indices exceeding those of BBR-M (bacteria: BPM/BBR-M = 4.20/3.47; fungi: BPM/BBR-M = 3.25/2.89).

**Figure 2:**
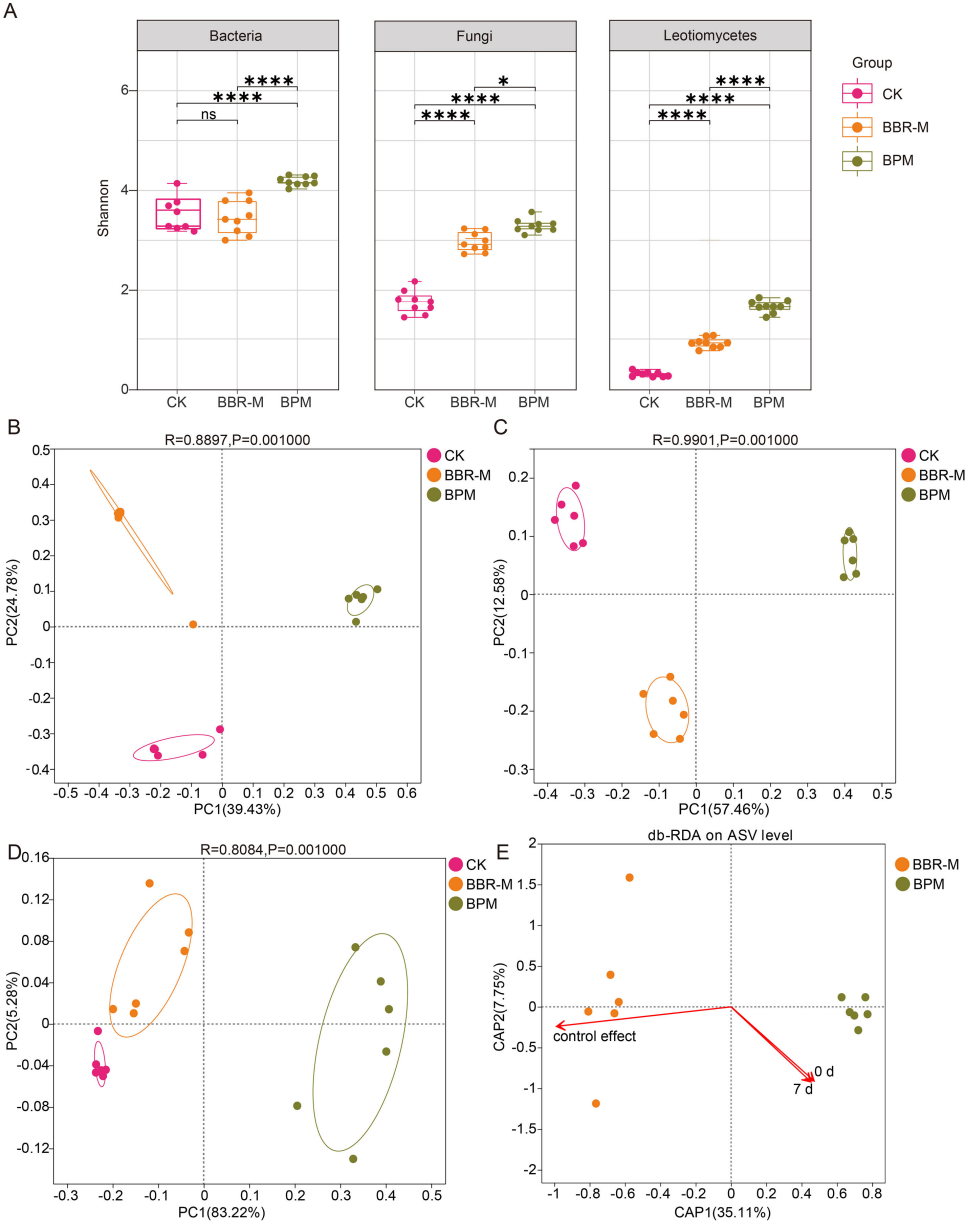
Analysis of microbial diversity of the strawberry phyllosphere. Treatments include control (CK), nano-berberine (BBR-M), and bupirimate (BPM). **(A)** Boxplots of Shannon diversity indices for bacteria, fungal and Leotiomycetes communities; **(B)** Principal coordinates analysis (PCoA) of bacterial communities based on Bray-Curtis dissimilarity at the ASV level; **(C)** PCoA of fungal communities based on Bray–Curtis dissimilarity at the ASV level; **(D)** PCoA of Leotiomycetes communities at the genus level. **(E)** Redundancy analysis (RDA) showing the relationship between powdery mildew control efficacy and disease index, with fungal community structure as the background.

Given that the causal agent of strawberry powdery mildew belongs to the fungal class Leotiomycetes, we further examined the alpha diversity of Leotiomycetes under different treatments. Both BBR-M and BPM significantly increased Leotiomycetes diversity compared to CK, and the effect was more pronounced under BPM (Shannon index: BPM/BBR-M = 1.53/0.85) (**Figure 2A**). Additional alpha diversity indices are presented in **Table 1**.

**Table 1:** Fungal and bacterial diversity indices of the strawberry phyllosphere under different treatments.

Microorganism	Sample name	Sequence numbers	Coverage/%	Alpha diversity			
				Sobs	ACE	Chao	Shannon
Bacteria	CK	48411	99.99	421.00±59.75bc	422.41±60.24bc	421.76±59.94bc	3.49±0.16b
	BBR-M	48411	100.00	453.17±29.28b	453.56±29.43b	453.30±29.33b	3.47±0.15b
	BPM	48411	99.98	635.67±34.62a	637.62±34.74a	637.19±34.69a	4.20±0.04a
Fungi	CK	42338	100.00	77.33±6.30b	77.72±6.39b	77.50±6.39b	1.65±0.12c
	BBR-M	42338	100.00	93.50±8.22b	93.90±8.25b	93.50±8.22b	2.89±0.09b
	BPM	42338	99.99	192.83±15.43a	193.58±15.42a	193.36±15.48a	3.25±0.07a

The results in the table are expressed as 'mean ± SE'. There was a statistically significant difference between different letter representation treatments (*p<*0.05, Duncan 's multiple range test).

Significant differences in the β-diversity of phyllosphere microbial communities were observed across fungicide treatments, including bacterial, fungal, and Leotiomycetes assemblages. Principal coordinates analysis (PCoA) based on Bray-Curtis dissimilarity revealed that the first two principal coordinates (PC1 and PC2) accounted for 39.43% and 24.78% of the variation among bacterial communities, respectively, explaining a cumulative 64.21% of the total variation. As shown in **Figure 2B**, samples from the BPM, BBR-M, and CK treatments clustered primarily in the first, second, the PCoA plot showed a clear spatial separation of samples according to treatment, with distinct clusters for BPM, BBR-M, and CK groups.

A comparable pattern was observed in the fungal community. PCoA based on Bray-Curtis distance showed that PCo1 and PCo2 explained 57.46% and 12.58% of the variance, respectively, accounting for 70.04% in total. An ANOSIM test further confirmed that fungal community structures differed significantly between treatments (R = 0.9901, *p* < 0.01) (**Figure 2C**).

Comparison of treatment-specific ASVs revealed that all fungicide treatments substantially increased the number of unique bacterial and fungal ASVs, with BPM resulting in a significantly higher number of specific ASVs than BBR-M (**Supplementary Figure 1**). Furthermore, PCoA analysis of Leotiomycetes communities demonstrated significant differences in community composition across treatments (**Figure 2D**).

#### 3.1.2 Correlation between fungal communities and powdery mildew indices

Different fungicide treatments were closely associated with variations in disease control efficacy against strawberry powdery mildew. Redundancy analysis (RDA) revealed a strong correlation between the structure of the phyllosphere fungal community and disease control. The first two constrained axes (CAP1 and CAP2) explained 35.11% and 7.75% of the total variation, respectively. Notably, the BBR-M treatment vector showed a geometric alignment with the control efficacy vector. In contrast, the BPM vector was oriented oppositely. From the perspective of vector projection, BBR-M also exhibited a longer projection on the control efficacy axis compared to BPM (**Figure 2E**).

#### 3.1.3 Bacterial community composition and relative abundance

**Figure 3:**
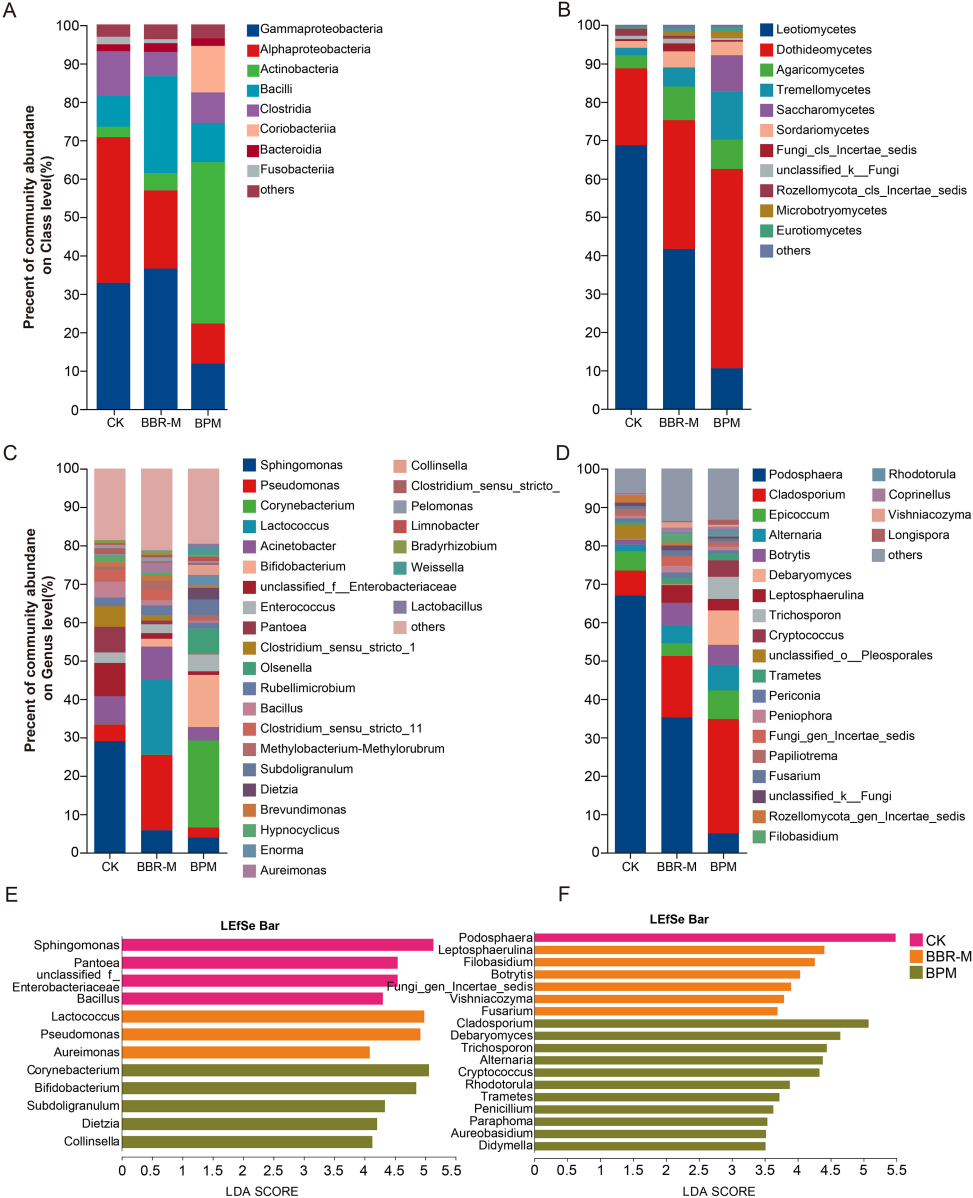
Microbial composition of the strawberry phyllosphere under different treatments. Treatments include control (CK), nano-berberine (BBR-M), and bupirimate (BPM). **(A)** Analysis of dominant species composition at the level of bacterial class; **(B)** Analysis of dominant species composition at the level of fungal class; **(C)** Analysis of dominant species composition at the genus level; **(D)** Analysis of dominant species composition at the fungal genus level; **(E)** Lefse analysis results of different groups of bacteria (LDA score > 4.0, p < 0.05); **(F)** Lefse analysis results of different groups of fungi (LDA score > 4.0, p < 0.05).

A total of 29530 high-quality bacterial sequences were obtained from 18 phyllosphere samples and clustered into 4724 amplicon sequence variants (ASVs). These ASVs were taxonomically assigned to 36 phyla, 106 classes, 988 genera, and 1613 species. At the class level, bacterial community composition was influenced by fungicide treatments, with several classes emerging or being substantially enriched. As shown in **Figure 3A**, the dominant bacterial classes across all treatments were Gammaproteobacteria (27.19%), Alphaproteobacteria (22.81%), Actinobacteria (16.38%), Bacilli (14.52%), Clostridia (8.51%), and Bacteroidia (2.07%).

Comparative analysis revealed significant treatment-specific shifts in class-level abundance. In the BBR-M group, the relative abundances of Gammaproteobacteria, Bacilli, and Bacteroidia increased by 3.71%, 17.06%, and 0.449%, respectively, all significantly higher than those observed in CK and BPM (*p <* 0.05). Conversely, Actinobacteria abundance was markedly elevated (by 39.1Coriobacteriia was highly enriched only in the BPM group, with a relative abundance of 12.07%, compared to 0% in both CK and BBR-M, indicating a distinct treatment-driven accumulation.

At the genus level, fungicide applications also led to substantial restructuring of bacterial community profiles, with distinct dominant taxa emerging in each group. Genera with cumulative relative abundance below 0.1% were excluded from analysis (**Figure 3C**). The most prevalent genera included *Sphingomonas* (12.91%), *Pseudomonas* (8.90%), *Corynebacterium* (7.58%), *Lactococcus* (6.56%), *Acinetobacter* (6.41%), *Bifidobacterium* (5.20%), and *unclassified Enterobacteriaceae* (3.70%).

Further comparisons revealed that fungicide treatments induced significant alterations in genus-level abundances. The relative abundance of *Sphingomonas* decreased substantially across all treated groups. In the BBR-M group, the abundances *Pseudomonas*, *Lactococcus*, and *Bifidobacterium* increased significantly compared to the control, by 15.31%, 19.58%, and 2.03%, respectively (*p <* 0.05). BPM treatment resulted in notable increases in *Corynebacterium* (22.61%), *Bifidobacterium* (13.56%), and *Olsenella* (6.50%) relative to CK. Compared with BBR-M, the abundances of *Bifidobacterium* and *Olsenella* under BPM treatment were also significantly higher, with increases of 11.53% and 6.50%, respectively *p <* 0.05).

#### 3.1.4 Fungal community composition and relative abundance

A total of 1452330 high-quality fungal sequences were obtained from 18 phyllosphere samples and clustered into 1434 amplicon sequence variants (ASVs). These ASVs were taxonomically assigned to 10 phyla, 34 classes, 456 genera, and 707 species. At the class level, fungal community structure was substantially reshaped by the different treatments, with notable shifts in the relative abundance of dominant taxa (**Figure 3B**). The most abundant classes across treatments were Leotiomycetes (40.28%), Dothideomycetes (35.15%), Agaricomycetes (6.60%), Tremellomycetes (6.46%), and Sordariomycetes (3.15%).

Statistical analysis indicated that the relative abundances of fungal classes differed significantly among treatments (*p <* 0.05). In particular, Leotiomycetes, which includes the powdery mildew pathogen, declined markedly following fungicide application. Its relative abundance decreased from 68.64% in the control group to 41.64% in BBR-M and 10.56% in BPM, representing reductions of 27.00% and 58.08%, respectively. These findings suggest that both BBR-M and BPM treatments were effective in suppressing pathogenic fungi within the phyllosphere, with BPM exhibiting a more pronounced reduction in Leotiomycetes abundance.

At the genus level, fungal communities were also strongly influenced by fungicide application. Genera with total relative abundances below 0.01% were excluded from the analysis. The most dominant genera across treatments were *Podosphaera* (35.70%), *Cladosporium* (17.43%), *Epicoccum* (5.25%), *Alternaria* (4.40%), and *Botrytis* (4.11%) (**Figure 3D**).

Fungicide application significantly altered the composition of fungal genera (*p <* 0.05). The relative abundance of *Podosphaera*, the primary powdery mildew pathogen, decreased in both treatment groups, indicating effective disease suppression. Meanwhile, the suppression of the previously dominant *Podosphaera* created ecological space for other pathogenic fungi, resulting in their relative enrichment. In particular, under BPM treatment, the relative abundances of *Cladosporium*, *Epicoccum*, and *Alternaria* increased by 23.29%, 2.51%, and 4.84%, respectively, compared to the control (*p* < 0.01).

### 3.2 Differential microbial taxa across treatments

Fungicide treatments resulted in distinct shifts in bacterial taxa that significantly contributed to the differentiation of microbial communities across samples. To identify key genera responsible for these differences, we applied linear discriminant analysis effect size (LEfSe) based on taxonomic composition data. As shown in **Figure 3E**, twelve bacterial genera with LDA scores greater than 4 were found to be significantly associated with treatment-specific effects (*p <* 0.05). Several beneficial genera such as *Sphingomonas*, *Pantoea*, and *Bacillus* were significantly enriched in the CK group.

In contrast, BBR-M treatment led to the enrichment of *Lactococcus* and *Pseudomonas* in the phyllosphere, while BPM treatment promoted the accumulation of *Corynebacterium*, *Bifidobacterium*, *Dietzia*, and *Collinsella*. These recruited beneficial taxa may contribute collectively to disease suppression and microbiome resilience under fungicide pressure.

Regarding fungal communities, the relative abundance of the powdery mildew pathogen *Podosphaera* was significantly reduced in both treatment groups. LEfSe analysis identified *Podosphaera* as highly enriched in CK, with an LDA score of 5.48, indicating its dominant contribution to community-level differences (**Figure 3F**). The substantial decrease in *Podosphaera* following treatment corresponded with relative increases in other fungal taxa, including both potential pathogens and known biocontrol agents.

In the BBR-M group, genera such as *Botrytis*, *Leptosphaerulina*, *Fungi_gen_Incertae_sedis*, and *Fusarium* showed significant enrichment. In the BPM group, *Cladosporium*, *Alternaria*, *Cryptococcus*, *Aureobasidium*, and *Didymella* were more abundant. In addition, several biocontrol fungi reported to be beneficial for plant health also increased in abundance, including *Filobasidium* and *Vishniacozyma* (BBR-M), and *Debaryomyces*, *Rhodotorula*, *Trichosporon*, *Trametes*, *Penicillium*, and *Paraphoma* (BPM).

### 3.3 Fungal community nutritional patterns and bacterial functional prediction

**Figure 4:**
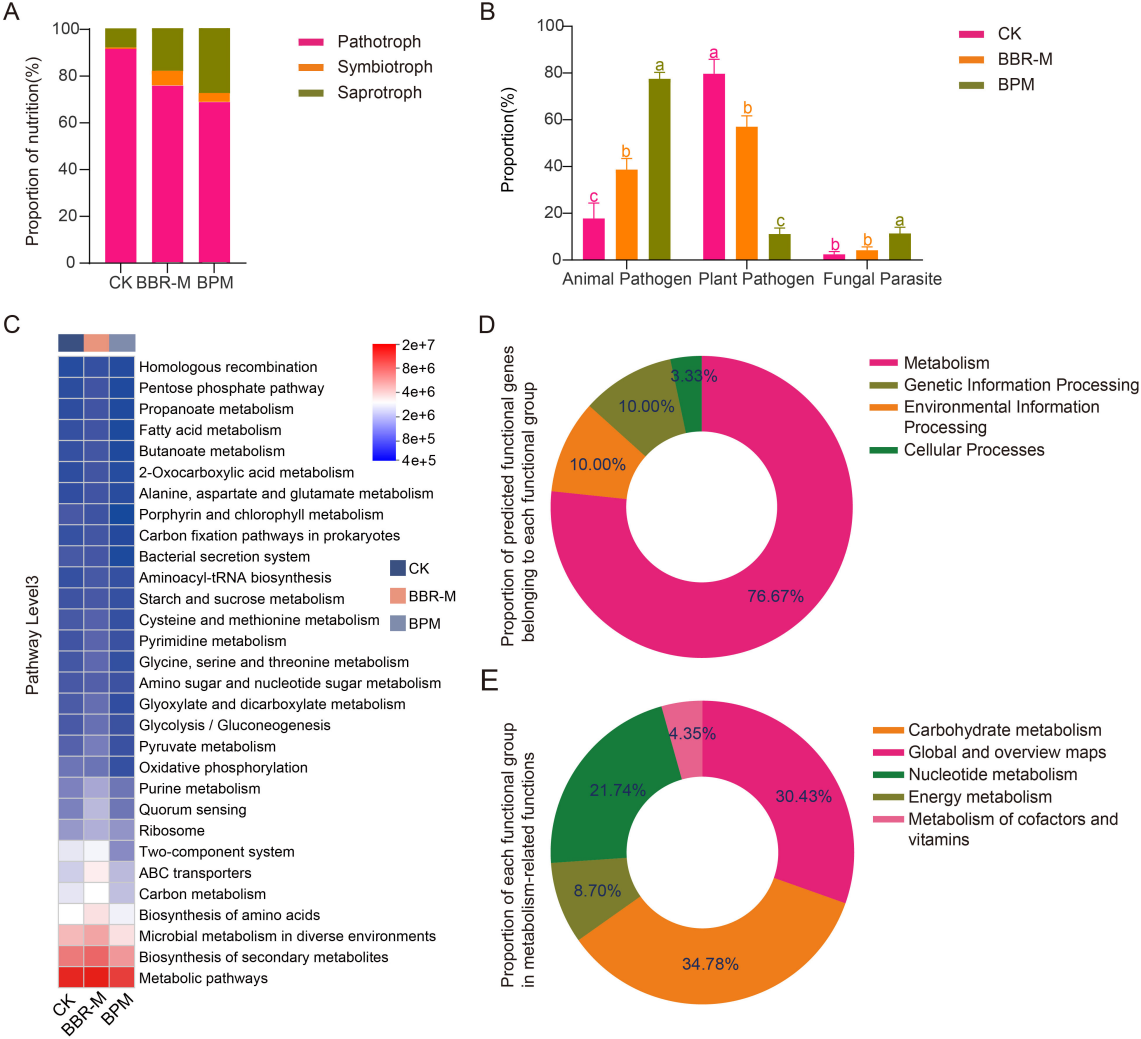
Prediction of fungal and bacterial functions in the strawberry phyllosphere under different treatments. Treatments include control (CK), nano-berberine (BBR-M), and bupirimate (BPM). **(A)** The relative abundance of fungal nutrition; **(B)** The relative abundance of Pathotroph, Symbiotroph and Saprotroph; **(C)** The relative abundance of bacterial KEGG Pathway Level3 heat map analysis; **(D)** The relative abundance of different functional groups was predicted; **(E)** The relative abundance of metabolic pathways.

Within the strawberry phyllosphere fungal community, plant pathogen was identified as the dominant functional group; however, its relative abundance declined most markedly following BPM treatment. In contrast, animal pathogen and fungal parasite groups exhibited notable increases under the same treatment. A total of 1214 fungal ASVs were identified, of which 960 were assigned to 58 trophic modes using the FUNGuild database. These trophic modes showed significant variation in relative abundance across treatments. In the control group (CK), pathotrophs were the most abundant trophic mode, while symbiotrophs were the least represented. After fungicide application, pathotroph abundance decreased significantly, whereas symbiotrophs and saprotrophs increased (**Figure 4A**).

Further analysis of the pathotroph subgroup revealed that plant pathogen was predominant, accounting for 85.05% in CK, followed by 69.02% in BBR-M, and 56.67% in BPM (*p <* 0.05). In contrast, Animal Pathogens increased steadily across treatments, from 15.15% in CK to 27.52% in BBR-M and 53.46% in BPM. Fungal parasites showed a significant rise only in the BPM group (8.01%), with no significant difference between CK and BBR-M (**Figure 4B**).

The predicted functional composition of strawberry phyllosphere bacterial communities varied significantly among fungicide treatments. Functional inference generated using PICRUSt2, based on 16S rRNA gene sequences, provided predicted functional potentials rather than experimentally validated functions. Most predicted bacterial genes were associated with four primary KEGG functional categories: metabolism, environmental information processing, genetic information processing, and cellular processes (**Figure 4C**).

Of the top 30 most abundant level 3 KEGG pathways, 80.00% were related to metabolism, 10.00% to environmental information processing, 6.67% to genetic information processing, and 3.33% to cellular processes (**Figure 4D**). Within the metabolic category, core functions such as general metabolic pathways, secondary metabolite biosynthesis, and microbial metabolism in diverse environments were consistently enriched across all treatments. A more detailed analysis of the top 15 functional pathways showed that carbohydrate metabolism had the highest relative abundance (34.78%), followed by global and overview maps (30.43%) and nucleotide metabolism (21.74%). Pathways related to energy metabolism and cofactor and vitamin metabolism were also represented, accounting for 8.70% and 4.35%, respectively (**Figure 4E**). These predicted functions suggest that bacterial metabolic activity may play an important ecological role in the phyllosphere and that its potential functional profile could be associated with fungicide application, although further experimental validation (e.g., metabolite profiling or qPCR for functional genes) is needed.

Further analysis revealed significant differences in several key metabolism-related pathways among treatments. For instance, the relative abundances of biosynthesis of amino acids (CK: 0.031; BBR-M: 0.033; BPM: 0.038), alanine, aspartate and glutamate metabolism (CK: 0.0069; BBR-M: 0.0071; BPM: 0.0082), and aminoacyl-tRNA biosynthesis (CK: 0.0074; BBR-M: 0.0076; BPM: 0.0095) increased significantly following fungicide application, with BPM treatments showing significantly higher levels than BBR-M. In pathways related to purine metabolism (CK: 0.013; BBR-M: 0.015; BPM: 0.016) and pyrimidine metabolism (CK: 0.008; BBR-M: 0.009; BPM: 0.010), the relative abundances also increased significantly after treat. For ABC transporters, relative abundance increased following treatment, with BBR-M and BPM showing increases of 0.008 and 0.004, respectively. The increase in the BBR-M group was significantly greater than that in the BPM group. In contrast, oxidative phosphorylation showed a consistent decline in relative abundance after fungicide application (CK: 0.012; BBR-M: 0.010; BPM: 0.010), with no significant difference between BBR-M and BPM. In addition, both two-component systems (CK: 0.027; BBR-M: 0.025; BPM: 0.018) and bacterial secretion systems (CK: 0.0087; BBR-M: 0.0076; BPM: 0.0070) were significantly downregulated following treatment. The abundance of the two-component system pathway was significantly lower in the BPM group compared to BBR-M, while differences in the bacterial secretion system were not significant.

### 3.4 Characteristics of phyllosphere microbial network

**Figure 5:**
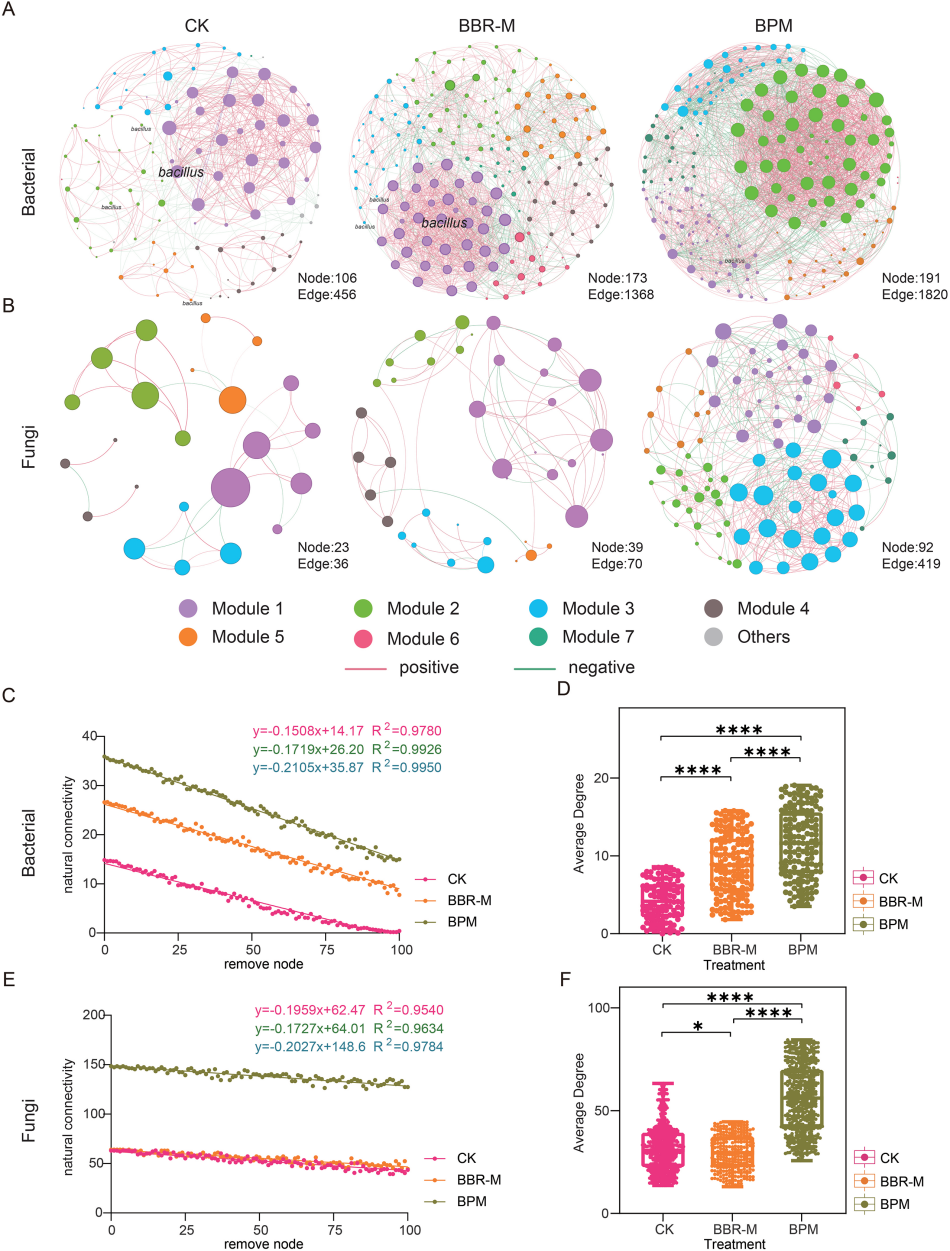
Co-occurrence networks and network properties of strawberry phyllosphere microbiota. Treatments include control (CK), nano-berberine (BBR-M), and bupirimate (BPM). Each node represents an amplicon sequence variant (ASV), with node size positively correlated with its weight. Node color indicates different modules, while edges represent correlations between ASVs. Red edges denote positive correlations and green edges indicate negative correlations. Networks were inferred using the SparCC algorithm and visualized based on correlations with |r| > 0.6 and p < 0.05. In panel **(A)**, nodes annotated as Bacillus represent ASVs taxonomically assigned to the Bacillus genus, highlighting their central position within the bacterial co-occurrence network under nano-berberine treatment. **(A)** Bacterial co-occurrence network; **(B)** Fungal co-occurrence network. (**C–F**) Comparisons of network stability and complexity: **(C)** bacterial network stability, **(D)** bacterial network complexity, **(E)** fungal network stability, and **(F)** fungal network complexity. Different colors represent different treatments. Asterisks indicate significance levels: “*” p<0.05; “***” p ≤ 0.0001. Effect size (Cohen’s d and effect size r) was calculated for comparisons with significant differences. For the average degree between CK and BBR-M, Cohen’s d = 0.225 and r = 0.11, indicating a small effect size.

To evaluate the effects of fungicide treatments on microbial interactions within the strawberry phyllosphere, co-occurrence networks were constructed using the SparCC algorithm. The resulting networks revealed markedly different topological structures across treatments, with significant alterations observed in both bacterial and fungal communities (**Figures 5A, B**).

In the bacterial networks, fungicide application significantly increased the number of nodes, edges, and the average degree compared to the control (**Figure 5D**). Similarly, fungal networks exhibited increases in nodes and edges. However, the average degree showed only a slight decrease under nano-fungicide (BBR-M) treatment relative to CK (**Figure 5F**). Statistical analysis indicated that the difference between CK and BBR-M was significant but characterized by a small effect size (Cohen’s d = 0.225, r = 0.11), suggesting that the observed variation reflects a limited biological impact rather than a substantial shift in network topology. BPM treatment led to a significant increase in average degree than BBR-M in both networks (*p <* 0.05), indicating a more complex microbial interaction network under BPM.

However, this increase in complexity did not necessarily translate to improved structural integrity. BPM treatment resulted in the lowest modularity values-0.36 for bacteria and 0.55 for fungi-and the greatest reduction in natural connectivity (**Supplementary Table 1**).

Interestingly, the effects on stability diverged between bacterial and fungal communities. In the bacterial network, BBR-M treatment led to decreased natural connectivity compared to CK, implying weakened network resilience. In contrast, the fungal network under BBR-M showed increased modularity and natural connectivity, reflecting enhanced structural stability. Among all treatments, BBR-M yielded the highest natural connectivity in the fungal network (**Figures 5C, E**).

Notably, members of the genus *Bacillus* were identified as one of the core bacterial taxa within the co-occurrence network, suggesting their potential ecological importance in maintaining phyllosphere microbial connectivity and stability. This finding provided a rationale for isolating a representative strain (*B. siamensis*) to further evaluate its antagonistic function.

### 3.5 Biocontrol efficacy of the core phyllosphere microbe *B. siamensis*

**Figure 6:**
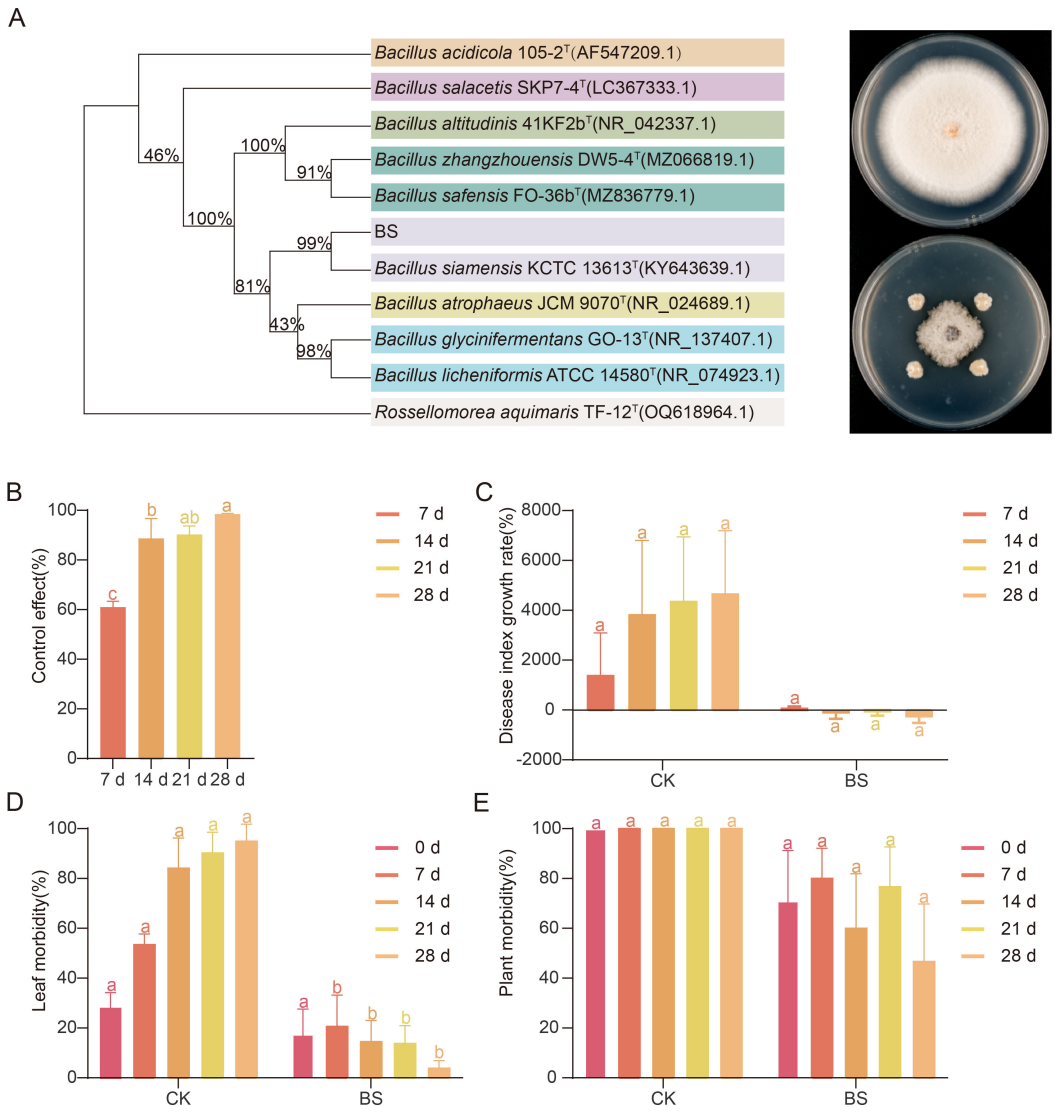
Antagonistic activity of the core phyllosphere bacterium Bacillus siamensis against major strawberry pathogens. **(A)** In vitro antagonism of B. siamensis (BS), isolated from the phyllosphere, against C. nymphaeae, the causal agent of strawberry anthracnose. The phylogenetic relationship of B. siamensis with closely related Bacillus species was inferred from 16S rRNA gene sequences using the maximum likelihood estimation in MEGA 11, with 1000 bootstrap replicates. **(B)** Field efficacy of foliar BS application for powdery mildew control, Disease index growth rate **(C)**, leaf-level disease incidence **(D)**, and plant-level disease incidence; **(E)** after BS treatment.

A bacterial strain was isolated from the leaves of strawberry plants treated with the nano-fungicide. Colonies were nearly circular, off-white, with smooth and opaque surfaces. The colony edges were irregularly wrinkled, the texture was mucilaginous, and the surface gradually collapsed over time, eventually forming a membrane-like structure. This strain was called BS. Molecular identification and NCBI BLAST analysis confirmed the strain as *B. siamensis*. Antagonistic assays against the common anthracnose pathogen *C. nymphaeae* demonstrated that BS exhibited strong inhibition, with an average inhibition rate of 79.22%, an average inhibition radius of 1.58 cm, and an average inhibition zone width of 0.22 cm (**Figure 6A**). When applied to strawberry plants naturally infected with powdery mildew in the field, BS showed progressive control efficacy, increasing from 60.99% at 7 d post-treatment to 98.18% by 28 d (**Figure 6B**). Although disease index growth rate and plant-level incidence did not differ significantly between BS and CK, leaf-level incidence showed a significant reduction starting at 7 d. After a brief increase of 3% immediately after application, leaf incidence under BS treatment declined by 16.76% from 7 d to 28 d, indicating a sustained and effective disease suppression (**Figures 6C–E**).

## 4 Discussions

This study presents the first multi-omics investigation of phyllosphere microbial community composition, diversity, and functional traits in response to nano-berberine and conventional chemical fungicide treatments in strawberry powdery mildew management. Our findings provide a theoretical foundation for microbial-assisted management of foliar diseases in strawberry. However, further advancement in quantitative microbiological techniques is required to accurately assess microbial abundance and resolve community dynamics under different treatment conditions. Such methodological improvements are critical for developing precision-targeted, ecologically sustainable disease management approaches.

### 4.1 Nano-fungicides and chemical fungicides both reduced powdery mildew but differed in effectiveness

Both the nano-fungicide and chemical fungicide effectively suppressed strawberry powdery mildew, but differed in their temporal performance and underlying mechanisms. Field experiments showed that by 28 d post-application, both nano-formulated berberine (BBR-M) and the systemic pyrimidine fungicide bupirimate (BPM) significantly reduced disease incidence and severity. The superior and more sustained performance of BBR-M was likely associated with its controlled-release behavior and high dispersibility, which facilitate prolonged retention and even distribution on the leaf surface. This interpretation is consistent with physicochemical characterization data showing a high Zeta potential (+24.4 mV) that may contribute to stable dispersion through electrostatic repulsion, and a higher decomposition temperature (215°C) suggesting enhanced thermal stability (Appah et al., 2019; Tian et al., 2022). Such properties are consistent with and may be associated with previously reported nano-formulations exhibiting improved stability and sustained release (Tang et al., 2021). In contrast, BPM rapidly inhibits pathogen energy metabolism via systemic action, enabling short-term efficacy. However, it lacks controlled-release properties and is more prone to environmental degradation, leading to suboptimal performance at early stages such as 7 d post-application.

Previous research reported that a 1:1000 dilution of bupirimate achieved 70.8% control of powdery mildew on the highly susceptible ‘Benihoppe’ cultivar at 7 d after the first spray. In this study, we applied a 1:216 dilution of bupirimate, a concentration commonly used in field practice. Differences in disease control outcomes may be due to multiple factors, including cultivar susceptibility (with ‘Monterey’ being moderately resistant and ‘Benihoppe’ more susceptible), initial pathogen load, site-specific fungicide history, and variation in absorption and systemic movement of the compound. These findings suggest the importance of considering fungicide characteristics, host genotype, and local agronomic conditions when designing disease management strategies.

Although a non-nano berberine control was not included in the field experiment, preliminary *in vitro* plate assays showed that the combination of bulk berberine and curcumin exhibited substantially weaker inhibition against *C. nymphaeae*, *Botrytis cinerea*, and *C. viniferum* than the nano-berberine formulation (BBR-M) (**Supplementary Figure 2**). These findings suggest that the enhanced antifungal efficacy observed in BBR-M treatment is likely associated with the nano-formulation process rather than the active compound itself. Nevertheless, we acknowledge this as a methodological limitation and plan to include a non-nano berberine control under the same pathogen system in future experiments to more clearly distinguish nano-specific effects.

### 4.2 Differential impacts of nano-fungicides and chemical fungicides on the diversity of strawberry phyllosphere microbiota

Plant leaves serve as the primary site of photosynthesis, playing a vital role in light absorption, nutrient synthesis, and the regulation of water and gas exchange, thereby sustaining plant health and ecological function (Li et al., 2022; Liu et al., 2020; Timm et al., 2018). The phyllosphere hosts diverse microbial communities, where higher microbial diversity is often linked to enhanced stability and resilience of the microbial ecosystem (Chen et al., 2020). It is well established that chemical fungicide application can significantly reshape the composition and diversity of leaf-associated microbiota-typically by suppressing phytopathogenic fungi and enriching beneficial bacterial taxa-thereby reducing disease occurrence (Siegieda et al., 2024; Vischetti et al., 2024). In our study, conventional fungicide treatment significantly enhanced both bacterial and fungal diversity and richness. In contrast, nano-berberine treatment did not induce significant changes in fungal diversity (*p <* 0.05), suggesting a relatively mild ecological impact on the phyllosphere microbiome compared to conventional fungicides. This finding is consistent with prior studies showing that some nanomaterials exert minimal interference with phyllosphere microbial balance (Ďúranová et al., 2024; Lowry et al., 2024; Wang et al., 2023).

### 4.3 Nano-fungicides and chemical fungicides differentially reshape co-occurrence networks in the strawberry phyllosphere

The application of nano-berberine (BBR-M) induced significant shifts in phyllosphere microbial community composition and interaction patterns. Bacterial richness markedly increased, which may be associated with the proliferation of r-strategist taxa and the enrichment of fungicide-metabolizing species capable of utilizing agrochemical compounds as nutrient sources (Yang et al., 2023). However, the concurrent decline in bacterial diversity suggests a community shift characterized by the increased dominance of several taxa, which may reflect potential changes in interspecies interactions or niche overlap (Louca et al., 2018). In contrast, fungal richness remained stable, while diversity increased, reflecting suppression of powdery mildew pathogens and the enrichment of fungal taxa associated with xenobiotic degradation, thereby enhancing overall fungal community heterogeneity.

At the network level, bacterial co-occurrence networks under BBR-M treatment exhibited increased structural complexity, as evident by higher node number, edge density, and average degree. However, these enhancements were accompanied by reduced network stability, characterised by a predominance of weak, non-specific interactions and a collapse of previously cohesive modules. The resulting topology was consistent with a scale-free network model, with connectivity disproportionately concentrated around a few highly connected hub taxa, which are typically associated with greater susceptible to targeted perturbations (Albert et al., 2000). In contrast, fungal networks under BBR-M treatment demonstrated increased modularity and natural connectivity, indicating improved structural organization and ecological resilience. The presence of distinct modular partitions and robust intra-module links suggest the emergence of a complex adaptive system capable of buffering external stressors (Wang et al., 2021).

While BBR-M enhanced apparent bacterial network complexity, it did so at the expense of topological stability. In contrast, the fungal co-occurrence network exhibited higher robustness and efficiency, suggesting a more ecologically sustainable restructuring. Bupirimate (BPM) also significantly altered microbial community structure, increasing both bacterial and fungal richness and diversity.

However, unlike nano-berberine, BPM treatment resulted in reduced modularity and natural connectivity across both bacterial and fungal networks. The disintegration of core modules and increased reliance on highly connected nodes rendered the networks structurally fragile and sensitive to node loss. Although BPM was associated with greater microbial richness, the associated network architectures were more fragmented and less resilient representing a form of “unstable complexity” (Albert et al., 2000).

These network characteristics were closely associated with the functional responses of microbial communities. Under BBR-M treatment, the relative abundance of symbiotrophs and saprotrophs increased, implying that enhanced fungal network modularity supported beneficial functional groups contributing to nutrient cycling and pathogen suppression. Meanwhile, the downregulation of bacterial oxidative phosphorylation and two-component systems pathways suggested that the weakened bacterial network may have reduced metabolic efficiency and stress responsiveness.

In contrast, the structural fragility and reduced modularity observed under BPM treatment coincided with a notable increase in animal pathogens and fungal parasites, indicating a potential disruption of ecological balance and an increased risk of opportunistic colonization (Gao M. et al., 2021).

Overall, both BBR-M and BPM altered the composition of the phyllosphere microbiota, but BBR-M better preserved the structural cohesion of the fungal network. This suggests that fungicide selection affects not only the core microbial taxa but also their interactions, and that maintaining a stable and complex microbial network may be critical for plant health.

### 4.4 Nano-fungicides and chemical fungicides enrich beneficial phyllosphere bacteria via distinct taxonomic pathways

Application of both nano-berberine (BBR-M) and chemical fungicide (BPM) treatments altered the abundance of beneficial bacterial taxa in the strawberry phyllosphere, albeit with distinct enrichment patterns. Common phyllosphere bacteria such as *Pseudomonas* (Sangiorgio et al., 2022; Siegieda et al., 2024), *Sphingomonas* (Anneberg et al., 2024), *Weissella* (Zhang et al., 2016), and *Bacillus* (de Moura et al., 2021; Zhang et al., 2024)were variably affected. In the bacterial co-occurrence network, *Bacillus* appeared as one of the central genera, implying a potentially key ecological role in the phyllosphere system. This observation guided the isolation of a representative strain from this genus for functional validation. Notably, BBR-M treatment significantly enriched *Pseudomonas*, *Lactobacillus*, and *Bifidobacterium*, while BPM treatment increased the relative abundances of *Corynebacterium*, *Bifidobacterium*, and *Olsenella*.

Although fungal pathogens decreased significantly 7 d post-treatment in both groups, disease control efficacy differed. BBR-M achieved superior field control, which may be associated with a more stable microbial network. In contrast, BPM-treated plots showed disease resurgence despite the early pathogen suppression, indicating that this pattern may be associated with microbial network instability and subsequent recolonization by powdery mildew pathogens.

Changes in the relative abundance of key beneficial bacteria also reflected niche shifts. *Sphingomonas* declined under both treatments, indicating poor adaptation to the altered phyllospheric environment. In contrast, *Pseudomonas* and *Lactococcus* emerged as dominant taxa following BBR-M application, while *Corynebacterium* became dominant in BPM-treated communities. These shifts highlight different ecological trajectories in microbial community reassembly.

Functionally, BBR-M-enriched communities relied more on nutrient competition and nucleotide-based signaling to inhibit pathogen colonization and improve plant stress tolerance, despite reduced capacities for environmental sensing. This pattern was associated with a stable yet competitive-symbiotic microbial network. In contrast, BPM-enriched communities enhanced antimicrobial metabolite production and nutrient cycling, but displayed lower protein biosynthesis and sensing capacity, weakening microbe–plant interactions and resulting in a simplified, less resilient network. These functional inferences align with co-occurrence network analysis, reinforcing that while BBR-M is associated with higher microbial diversity and ecological stability, its bacterial network remains structurally fragile. BPM, by contrast, was associated with a more complex but highly vulnerable network with increased reliance on a few keystone taxa.

### 4.5 *B. siamensis* exhibited high efficacy in controlling strawberry powdery mildew

Given its central position in the bacterial co-occurrence network, we hypothesize that *Bacillus* plays an important ecological role in the phyllosphere. Therefore, we isolated the representative strain *B. siamensis* from the phyllosphere of strawberries treated with nano-berberine to further examine its antagonistic potential and explore the functional implications of community changes.

In this study, a strain of *B. siamensis* with strong antagonistic activity against *C. nymphaeae*, a major causal agent of strawberry anthracnose, was successfully isolated from strawberry leaves collected from nano-berberine-treated plants. Members of the genus *Bacillus* were identified as core taxa within the BBR-M phyllosphere co-occurrence network, suggesting a potential ecological association rather than a direct causal relationship between BBR-M application and *Bacillus* enrichment. Originally identified in 2010 (Sumpavapol et al., 2010), *B. siamensis* is recognized as a valuable biocontrol resource due to its ability to produce a variety of antifungal compounds (Ni et al., 2023), enhance defense-related enzyme activities in rice (Sahu et al., 2020). Research on this species remains at an early stage, with most studies focusing on strain isolation, taxonomic identification, and antifungal efficacy. Previous findings have shown that *B. siamensis* effectively reduces the postharvest disease index in mango (Jiang et al., 2022), inhibit sugarcane smut and tobacco brown spot (Gao C. et al., 2021; Xie et al., 2021), and mitigate gray mold infection (Petrasch et al., 2019; Wang C. et al., 2022). Moreover, it has been reported to inhibit the growth of several major *Fusarium oxysporum*, *F. solani*, and *Phoma herbarum* (Fan et al., 2016). Together with its observed association with the BBR-M network, these findings suggest that *B. siamensis* may be associated with a potentially functional roles within the strawberry phyllosphere microbial network. Nonetheless, the present observations are correlative, and further validation—such as metabolite profiling or functional gene expression assays—will be required to confirm these ecological roles under field conditions.

*Bacillus* species, as naturally occurring bacteria, are frequently found within phyllosphere communities that enhance microbial network stability and suppress plant diseases (Chen et al., 2020). Utilizing such beneficial microorganisms to reduce reliance on synthetic fungicides and minimize fungicide residues has become an important research focus (Wang S.-Y. et al., 2022). Therefore, the scientifically guided and rational application of microbial agents such as *B. siamensis* holds great promise for reducing chemical fungicide use and promoting sustainable plant protection.

## 5 Conclusion

Collectively, our findings demonstrate that nano-fungicides and chemical fungicides exert distinct influences on the strawberry phyllosphere microbiota. Although both treatments altered microbial community composition and structure, their effects on diversity and network dynamics diverged substantially. Nano-fungicide application induced minimal disruption to the phyllospheric environment, whereas chemical fungicides elicited more pronounced ecological shifts.

Specifically, nano-fungicides preserved the structural stability of fungal communities, while bacterial networks exhibited decreased resilience-a pattern also observed in both microbial domains under chemical fungicide treatment. Importantly, both fungicide types effectively reduced the relative abundance of phytopathogens and facilitated the enrichment of beneficial microorganisms.Among these, *B. siamensis*, a core phyllosphere bacterium isolated from nano-fungicide-treated leaves, exhibited strong antagonistic activity against powdery mildew in field applications, underscoring its potential as a biocontrol agent.

These results support the preferential use of nano-fungicides in strawberry powdery mildew management, as they not only confer disease suppression but also promote ecological compatibility by maintaining microbiome stability and functional integrity in the phyllosphere.

## Data Availability

The datasets presented in this study can be found in online repositories. The names of the repository/repositories and accession number(s) can be found in the article/**Supplementary Material**.
