# Supplementary material for: Phyllosphere microbiome responses to nano-berberine and chemical fungicides in powdery mildew infected strawberry
Source: Front Plant Sci. 2025 Dec 8;16:1712123. doi: 10.3389/fpls.2025.1712123 (PMC12719446; doi:10.3389/fpls.2025.1712123)
Supplement: Supplementary file 1 [file DataSheet1.pdf]

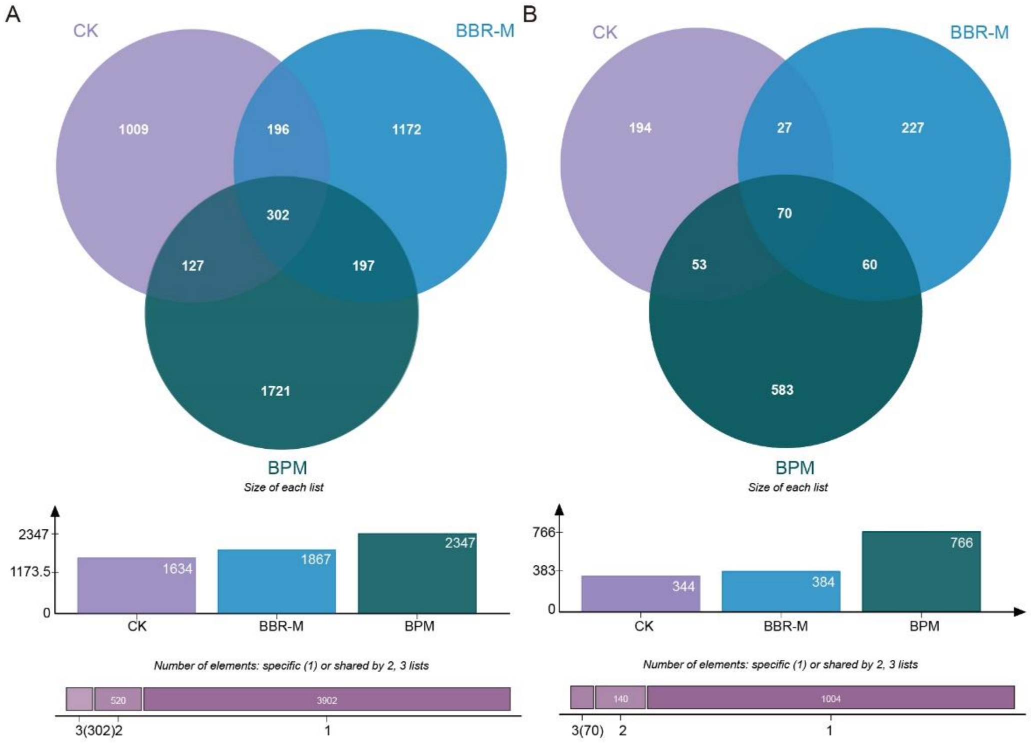

Supplementary Figures 1 Venn diagram of the ASV level

Supplementary Table 1 The properties of co-occurrence network graph

Microorganism	Sample name	Modularity	Connectivity	Clustering	Diamenter
Bacteria	CK	0.69	3.28	0.55	8.00
	BBR-M	0.52	2.67	0.51	6.00
	BPM	0.36	2.61	0.51	6.00
Fungi	CK	0.60	2.91	0.69	6.00
	BBR-M	0.62	4.46	0.52	9.00
	BPM	0.55	2.88	0.46	6.00

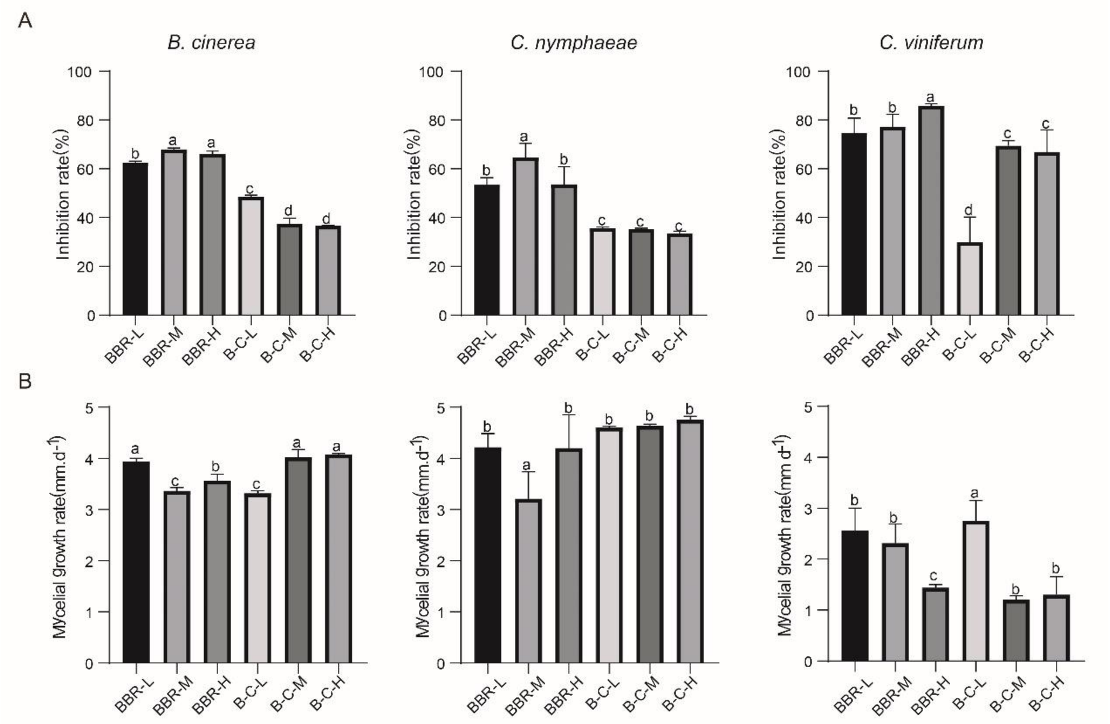

Supplementary Figures 2 In vitro antifungal activity of nano-berberine (BBR) and bulk berberine – curcumin formulations against major strawberry pathogens

Treatments include nano-berberine at three concentrations: BBR-L (2000 ppm), BBR-M (2500 ppm), and BBR-H (3000 ppm), and bulk berberine – curcumin formulations at corresponding concentrations: B-C-L (2000 ppm), B-C-M (2500 ppm), and B-C-H (3000 ppm), with a berberine-to-curcumin ratio of 2:1 (m/m). Data are presented as mean ± SD (n = 3). Different lowercase letters above bars indicate statistically significant differences among treatments ($p < 0.05$, Duncan's multiple-range test).

(A) Inhibition rate (%) of *B. cinerea, C. nymphaeae*, and *C. viniferum* under different treatments.

(B) Mycelial growth (mm) of the three pathogens under the corresponding treatments.
